# "Before womb to tomb" Yala health policy evaluation

**DOI:** 10.1186/1471-2458-14-S1-P2

**Published:** 2014-01-29

**Authors:** Sawat Apiwachaneewong, Plernpit Pongparinyagun, Tassanee Somsaman, Wanna Buri, Hameenaoh Sudwilai

**Affiliations:** 1 Yala Provincial Health Office, Yala, Thailand

## Background

Yala, the southernmost province of Thailand, is afflicted by the continual unrest. The health status needs to be improved, thus policy of Yala Healthy Life “From Womb to Tomb” was launched in 2011. In 2012, it was modified to “Before Womb to Tomb with Dignity by the Year 2016.” It focused on the root cause of anaemia of pregnancy, starting from the beginning of life in the womb. Female teenagers were prepared to be healthy child-bearing mothers. Consequently, the person should grow up to be a healthy adult and elderly. In the last period they should receive palliative care and at the end of life they will pass away with dignity. The objectives were to evaluate the policy, to explore occurring barriers and to investigate valuable suggestions in order to adjust the implementation during the next 3 years.

## Materials and methods

This was a half-policy formative evaluation by CIPP model (context, input, process, product), which employed documentation analysis, open-ended questions, discussion and site visit for obtaining data. The sample included health professionals, and representative committee of Yala people.

## Results

Results showed that the policy completely took care of people throughout the life cycle. There were efficient resources which supported the policy. After 2 years, most women's health status had slightly improved; maternal death rate dropped to zero, which had previously achieved for 10 years prior to the policy. It was found that the emerging barriers were lack of understanding in the policy and the violent situation in Yala made outreach difficult. Results suggested that some details should be adjusted appropriately to suit the culture and life style.

## Conclusions

The trend towards future success is feasible and going in the right direction. This is because in the fiscal year of 2013, Thailand Ministry of Public Health (MOPH) has announced health care strategies by age groups, same concept as the policy of Yala, which assures that the goal of good health for the people of Yala is achievable.

